# Exploring hope and expectations amidst the shadows: Navigating through the hearts of cancer patients admitted to a palliative care unit

**DOI:** 10.1017/S1478951524002165

**Published:** 2025-01-21

**Authors:** Maria Nikoloudi, Florian Thanasko, Ioanna Tsatsou, Alexandra Mantoudi, Kyriaki Mystakidou

**Affiliations:** 1 Pain Relief and Palliative Care Unit, Department of Radiology, Aretaieion Hospital, School of Medicine, National & Kapodistrian University of Athens, Athens, Greece; 2 School of Medicine, National & Kapodistrian University of Athens, Athens, Greece; 3 Department of Oncology, Hellenic Airforce General Hospital, Athens, Greece; 4 Department of Nursing, University of West Attica, Athens, Greece

**Keywords:** Cancer, expectations from a palliative care unit, hope, interpretative phenomenological analysis, holistic care

## Abstract

**Objectives:**

The objective of this study is to conduct an in-depth exploration of the psychological well-being, hope, and expectations of cancer patients receiving care in a palliative care unit, utilizing a qualitative research approach.

**Methods:**

We employed the methodology of interpretative phenomenological analysis (IPA). Our data collection involved conducting 1-hour semi-structured interviews with the patients. In the subsequent data analysis, we applied investigator triangulation to ensure rigor and reliability.

**Results:**

Understanding patients’ hope and expectations from palliative care is crucial as it can serve as an indicator of the quality of care and motivate care providers to fulfill these expectations as much as possible. Throughout the IPA, 3 superordinate themes emerged from the qualitative data: cancer diagnosis and the spectrum of emotions, hope and cancer patient, and oncology patient expectations of palliative care.

**Significance of results:**

From the patient’s perspective, making sense of their cancer experience involves managing symptoms, redefining their understanding of illness, adapting to functional changes, and fostering open communication among themselves, their families, physicians, and the palliative care team. This underscores the crucial necessity for an interdisciplinary approach and emphasizes the importance of reinforcing positive support systems. In essence, our study delves into the multifaceted psychological aspects of cancer patients in the context of palliative care, shedding light on their hope and expectations as they navigate the challenging terrain of cancer treatment and palliative support.

## Introduction

After 60 years since the renowned psychiatrist Karl Menninger (1969) challenged the scientific community to recognize hope as a crucial yet often overlooked aspect of clinical practice, its significance continues to warrant greater acknowledgment from health-care professionals. Hope has been positively associated with health and plays a significant role in managing illness and the losses that accompany it (Nekolaichuk et al. 1999). In oncology, hope is essential for patients’ effective adaptation to the disease and a strategy for dealing with physical and psychological disruptions. It serves as a protective factor against the physical, psychological, and social burdens of illness (Berendes et al. 2010; Herth 1992). While the loss of hope is associated with reduced functionality and is linked to depression and anxiety, hope as a multidimensional concept, encompasses much more. It includes emotional, cognitive, and behavioral dimensions, extending beyond mere optimism to include patients’ aspirations for quality of life, dignity, and meaning in the face of illness (Pleeging et al. 2022; Rustøen 2021).

In oncology and palliative care, hope promotes disease management, psychological adaptation, and overall quality of life (Berendes et al. 2010). Despite its importance, gaps remain in understanding the interplay between hope, patient expectations, and care quality, particularly in sociocultural contexts such as Greece. In Greece, palliative care is influenced by social, economic, and cultural factors, including financial challenges. Focusing on Greece, we can identify strategies and best practices that may be applicable in other contexts facing comparable challenges. Greek culture emphasizesfamily-centered values and traditions, with families playing a significant role in caregiving due to the absence of a specific legal framework for palliative care until recently. This reliance burdens families and affects patients’ coping. Additionally, the insufficient training for health-care professionals in palliative care worsens these challenges, impacting care quality and patient experiences. Despite recent legislative efforts, implementation remains pending, highlighting persistent challenges within Greece’s health-care system.

Due to the complexity of hope’s impact on cancer patients and patients in general, its exploration has been and continues to be a challenge for many researchers (Feldman and Corn 2023). Hope and patient expectations are subjective concepts that depend on various factors, such as the individual’s personality, the truth about the disease, the prognosis, and the course of the disease, as well as knowledge of the concept and the provisions of palliative care (Ghandourh 2016).

Investigating hope and expectations in palliative care is fundamentally important as it can serve as an indicator of care quality and motivate providers to meet these expectations as much as possible. The functional value attributed to hope and expectations by many scientists makes further investigation imperative. The precise goal of this study is to examine hope and expectations among palliative care patients, focusing on their expectations of care. The study aims to understand how these factors influence care quality perception and how providers can better meet these expectations. Specifically, the research seeks to understand how hope and patient expectations shape the experiences of care within Greece’s sociocultural context. Expected outcomes include insights that could inform policy changes, training programs, and interventions to address patients’ emotional and psychological needs. By addressing gaps in prior research, the study aims to contribute to the broader understanding of hope’s functional value in palliative care and its potential to enhance care delivery and patient outcomes.

## Methods

### Phenomenology

Phenomenology delves into subjective experiences, aiming to uncover their essence. Individuals construct reality through mental frameworks. Building on Husserl’s perspective of intentional consciousness (Husserl 2019), this study emphasizes how perception and interpretation are subjective, revealing how individuals engage with experiences and construct reality. Husserl’s concept of intentionality – the directedness of consciousness toward objects or experiences – aligns with interpretative phenomenological analysis (IPA) by providing a theoretical foundation for exploring participants’ lived experiences and the meaning they ascribe to them.

### Participants

Six cancer patients participated in this study at Athens’ only public palliative care unit (PCU). The group consisted of 4 women and 2 men, all of whom met the inclusion criteria: a histologically confirmed cancer diagnosis, age 18 or older, proficiency in Greek, the ability to communicate effectively, and provision of written consent. Patients with impaired cognitive function were excluded from the study. The choice of 6 participants aligns with the principles of IPA, which emphasizes depth and detail over breadth, allowing for a nuanced exploration of individual experiences (Smith et al. 2009). This sample size is adequate to generate rich and meaningful data, balancing feasibility with the need to capture diverse perspectives. To maintain anonymity, pseudonyms were used.

### Data collection

**Figure 1.**
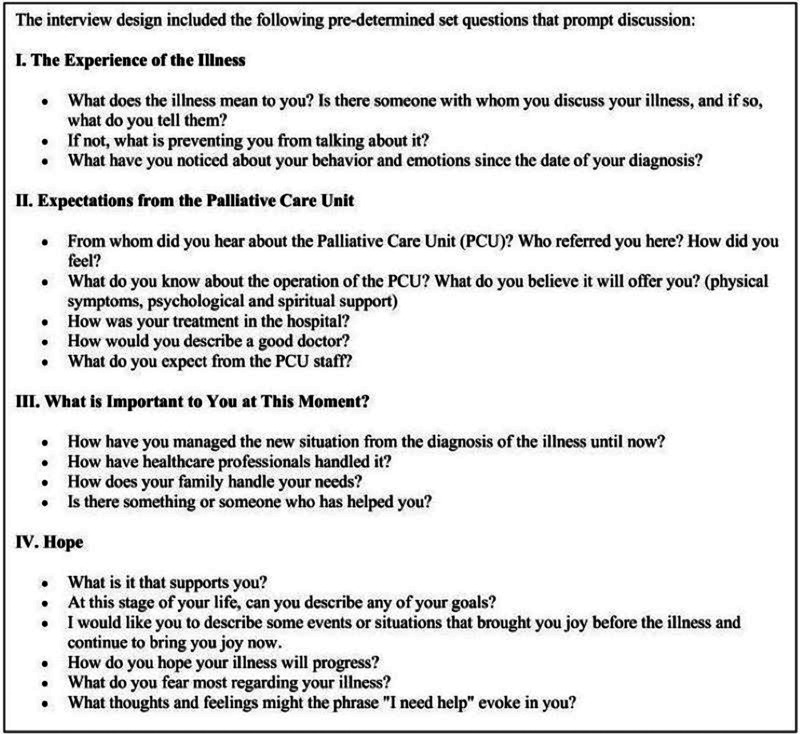
Pre-determined questions guiding the interview.

The university hospital ethics committee approved the study, and detailed demographic data were collected including gender, age, marital status, and education (Table 1). Participants voluntarily agreed, receiving instructions, signing consent forms, and given the research protocol. The principal investigator, a doctor with a PhD in Palliative Care, conducted semi-structured interviews during the patients’ first visit to the PCU (Figure 1). Conducting interviews at the first visit enabled capturing initial impressions and unfiltered experiences. The interviews were audio-recorded, transcribed, and translated into English for analysis and potential publication.

**Table 1.** Demographic and disease related characteristics of research participants (*n* = 6)

Mean age	60.3 years
Minimum age	53 years
Maximum age	76 years
Sex	
Female	4
Male	2
Family status	
Married	5
Unmarried	1
Level of education	
Primary school	1
High school	3
University	2
Type of cancer	
Breast cancer	2
Lung cancer	2
Bladder cancer	1
Ovarian cancer	1

n: number.

### Data analysis

The study utilized IPA. The principal investigator collaborated with 2 independent researchers, psychologists experienced in IPA, to enhance the study’s rigor.

The analysis followed a structured process. Interviews were transcribed verbatim and read multiple times to ensure familiarity with the data. Initial notes and reflections captured descriptive, linguistic, and conceptual elements of participants’ narratives. Emergent themes were then identified by examining patterns and connections within and across transcripts. These themes were subsequently grouped into higher-order categories to create a coherent thematic structure. To ensure credibility, investigator triangulation was employed; each researcher independently analyzed the data, and discrepancies were resolved through discussion, enhancing the reliability and validity of the results (Smith et al. 2009).

## Results

The results (Table 2) demonstrate triangulation. For reliability, the percentage of theme occurrences was set at over one-third of participants (Smith et al. 2009). In this study, almost all subordinate themes are observed at 100%, except for 1, expectations of psychological support, that appears at 83.32%, while the resulting superordinate themes are fully representative of the participants’ data. This fact implies an extremely high degree of reliability and validity.

**Table 2.** Superordinate themes and subordinate themes

Superordinate Theme 1: Cancer diagnosis and the spectrum of emotions	Superordinate Theme 2: Hope and cancer patient	Superordinate Theme 3: Oncology patient expectations of palliative care
Subordinate Themes		
Lightning in Ethria – The unexpected mandate.	Coping with the present and uncertainty	Distrust and traumatic experiences toward and from health-care professionals in the public hospital contrasted with positive experiences.
Fear	Navigating pain, symptoms, and the future	Referral and accessibility issues in PCU – Expectations from the PCU: I’m waiting to see. PCU as an unknown place
Anger and mourning: A 2-sided coin.	Relationships and the impact of Illness	Information expectations from the PCU Expectations of psychological support. Expectations of collaboration and building trust with medical staff.

### Cancer diagnosis and the spectrum of emotions

The first superordinate theme examines the emotional journey of diagnosis, spanning shock, numbness, fear, and ending with anger and sorrow, emphasizing the emotional aspect.

### Lightning in Ethria – The Unexpected Mandate

The diagnosis abruptly shifts patients from health to illness, causing shock and numbing. It’s an unexpected disturbance, like a lightning bolt, in their peaceful lives.

“Lightning in Ethria.” (Polenia, interview 1)

The diagnosis is a brief, challenging moment where participants feel trapped in frozen emotions, waiting for it to pass quickly. The diagnosis halts time, shattering life and leaving one feeling powerless and drowned in sorrow. Illness news disrupts the once-healthy self.

### Fear

The cancer diagnosis suppresses all emotions, leaving fear to dominate. Fear isn’t just an immediate response; it’s a future-oriented, protective emotion. The initial reaction is often freezing, like a mental pause.

“I’m afraid of what awaits me. Painful.” (Elena, interview 2)

Cancer is an unsettling event that strikes blindly, causing chaos and disarray. Patients are never prepared for how it unfolds, and its impact is grief and agony. Cancer is a constant, invisible source of shock and lasting damage. Participants live in perpetual fear of its unpredictable attacks, uncertain about the future with unanswered questions.

### Anger and mourning: A 2-sided coin

After the overwhelming fear, comes anger. In the initial phase of realizing the loss of health and the shattered life, intense anger arises.

“When I found out, of course, my reaction wasn’t what it should have been. I started hitting my hands against the wall. The medicine exists, don’t lie to me, you just don’t provide it because it’s a matter of money.” (Maria, interview 5)

Nervousness, trembling, and bodily drama manifest as an outburst, reflecting the struggle to accept cancer. This internal pressure finds release through aggression like kicking or hitting, serving as a relief valve. Maria exaggerates denial of her condition and distrusts health-care professionals. Her anger flows like a powerful river, warping reality yet offering an avenue for release. Rush of anger and mourning collide. Anger, relentless and uncontrollable, dances with denial, while mourning anticipates losses. All participants share similar reactions.

## Hope and cancer patient

The second superordinate theme delves into hope within the context of cancer, exploring patients’ unique experiences, emotions, and coping mechanisms.

### Coping with the present and uncertainty

Cancer deeply impacts individuals, shaping their experiences and adaptation based on factors like type, severity, and life stage. Post-diagnosis, the desire for normalcy clashes with economic and existential insecurities.

“They say, don’t stay at home, find ways out (…) Where should I go (…) anything I do to express myself is not for free.” (Polenia, interview 1)

Polenia struggles with unrealistic advice and financial constraints, while Magda feels the burden on loved ones. Elena fears losing her autonomy and dignity, emphasizing the importance of maintaining a basic quality of life.

“Cancer is a disease that you know has an end. (…) you will be happy if the next day is at least like the previous one and it hasn’t gotten worse.” (Elena, interview 2)

Participants mourn their precancer selves, facing daily uncertainty, anxiety, and a gradual decline in strength and roles. Yet, they strive for empowerment, viewing cancer as a conquerable enemy and holding onto hope for a better future. Despite the challenges, they seek relief from loneliness and the fear of dying alone, aiming for independence, freedom, and a redefined balance in life.

### Navigating pain, symptoms, and the future

Cancer patients prioritize immediate pain relief and peace in death, battling anxiety from chronic pain and treatment side effects. Pain, once protective, now signifies suffering and trauma, leading to fatigue, resignation, and a declining quality of life.

Polenia struggles to accept her condition due to the absence of pain, clinging to an unrealistic hope of preserving her old self.

“I feel very well. I don’t feel sick. I don’t feel anything, I don’t have anything, how is there a battle raging inside me?.” (Polenia, interview 1)

Hope centers on short-term goals and managing daily life, as the fear of relapse and pain looms.

The desire for a “good death,” ideally at home, reflects a need for dignity and peace.

“However, along with all this, I also had a very nice arrhythmia in my heart, and I said this might be a gift because if the heart doesn’t hold up, I will leave, so I won’t worry about the pain. I will just leave.” (Magda, interview 6)

Patients, exhausted from fighting, seek rest and focus on a quiet, gradual withdrawal from life.

### Relationships and the impact of illness

Illness unites and divides, reshaping relationships and hope. Petros emphasizes the importance of discussing the disease, while family and friends offer crucial, though sometimes strained, support.

“It’s something different, something that needs to be pushed more into the world. This cancer story has made me talk about my illness.” (Petros, interview 3)

Illness can lead to role shifts within families, causing denial, guilt, and frustration. Elena feels torn between her needs and her husband’s kindness, struggling with guilt. As hope evolves, patients balance their own needs with those of loved ones, navigating the emotional complexities of their condition.

## Oncology patient expectations of palliative care

The concept of expectations pertains to the likelihood that a behavior will result in a specific outcome, whether successful or unsuccessful (Atkinson 1957). What individuals expect is closely tied to their personal efforts to achieve the desired result. To meet their expectations, individuals need to understand which actions will lead to the desired outcome and have confidence in their self-efficacy (Bandura 1977).

### Distrust and traumatic experiences toward and from health-care professionals in the public hospital contrasted with positive experiences

Distrust in the medical field often stems from poor communication and a perception that financial interests outweigh patient care. This distrust leads some patients to seek urgent treatment and consider private health care, exposing public system flaws. Polenia, for example, experienced a lack of compassion from a doctor, damaging her trust. Elena and Magda also express frustration and distrust due to unaddressed concerns and procedural challenges.

“Unfortunately, I will always leave my doctor’s office very angry. I’m telling you, it’s like a red flag.” (Magda, interview 6)

Conversely, some patients report positive experiences when medical staff engage in transparent communication. For Maria, just having caring staff present was comforting. Petros appreciated clear communication that met his needs, easing his anxiety. Positive interactions, restore trust in medical professionals.

### Referral and accessibility issues in PCU – expectations from the PCU: I’m waiting to see. PCU as an unknown place

Many patients, like Polenia, Magda, and Elena, discovered PCU incidentally, seeking relief and emotional support.

“How did you feel when she suggested it to you?” (Principal investigator)

“I felt relief because I had another weapon beside me, and when I came, I was very satisfied because I felt that you listened to me completely, to my entire history, with great kindness (she cries), something that is missing. This is what I wanted to share.” (Elena, interview 2)

While initially resistant and skeptical, they found comfort in being listened to and valued the kindness they experienced. However, doubts about PCU’s purpose persisted, especially among those referred by oncologists, who feared gaps in care. Andreas felt abandoned but still trusted his oncologist.

“Where do you believe we will help you?” (Principal investigator)

“I don’t know, I don’t know, I will wait and see.” (Andreas, interview 4)

Expectations of PCU were unclear, with patients like Petros seeking stability and support,

“Stability. I like the fact that I won’t have a problem with the medication, that we will have a conversation, and if I need something, you will provide it.” (Petros, interview 3)

while others focused on pain relief and emotional connection to combat fear and isolation.

“What do you expect from us? (Principal investigator)

Your help and being able to talk about the pain and in general, to talk, it does me good. I enjoyed it.” (Maria, interview 5)

### Information expectations from the PCU

Patient satisfaction at the PCU hinges on receiving honest, clear information.

Patients want to be active decision-makers and expect transparency from the staff about treatments and possible relapses.

“I mean, I don’t want some doctors who talk behind the patient’s back. It’s me; you put me in the operating room, you cut me. Me! I want to know. (.)” (Maria, interview 5)

However, complex medical details and limited time often hinder understanding and lead to mistrust.

“What more did you want?” (Principal investigator)

“I wanted more time because he doesn’t know me.” (Magda, interview 6)

Some patients, like Polenia and Maria, have experienced trauma due to poor communication, while others, like Magda, prefer more compassionate care. Patients desire direct, transparent communication without intermediaries, believing they can handle the truth and expect the PCU to respect their need for knowledge.

### Expectations of psychological support

Patients seek psychological support to cope with their illness, express emotions, and alleviate loneliness. They view this support as essential for both mental and physical relief. Andreas struggles with sadness when hearing about others’ happiness, reflecting his own grief.

“When I hear something happy, for example, I feel sad.’’ (Andreas, interview 4)

Maria finds both growth and distress in her pain, while Magda seeks guidance on how to inform her child about her illness.

### Expectations of collaboration and building trust with medical staff

Patients seek trust, open communication, and collaboration with medical staff. They value the ability to ask questions, participate in decisions, and receive continuous care. Polenia and Petros emphasize the importance of feeling heard and supported by their oncologists.

“How would you describe a good health-care professional?” (Principal investigator)

“Someone we can talk to. For example, euthanasia has crossed my mind (.) I agree with euthanasia.” (Petros, interview 3)

Discussions about death are often avoided due to cultural taboos, complicating trust-building. However, open communication fosters a sense of belonging and security.

“To whom should I say things? I say them to someone who knows and can help you. If I tell my husband, what should I do? What will he tell me? I am disappointed because I would expect a more holistic approach.” (Elena, interview 2)

“Doctors are the soul of the hospital; they have to inspire confidence in you. You must have inspired confidence, and here I am, and there must be cooperation between us. I am ready to talk to you about anything.” (Petros, interview 3)

Patients expect transparency, stability, and hope from their care providers, seeing these as vital to managing their illness and easing distress.

In summary, the findings highlight that cancer patients face emotional turmoil, desire hope and control over their lives, and expect clear communication and psychological support from health-care providers. There is a strong emphasis on trust, transparency, and cultural sensitivity in palliative care to meet patients’ informational, emotional, and relational needs.

## Discussion

### Emotional responses to cancer

Emotions significantly influence how patients perceive life-threatening situations and their interactions with physicians during diagnosis and treatment decisions. Understanding both prominent and subtle emotions is vital for improving care.

In this study, Cancer patients commonly experience intense negative emotions, particularly fear, which persists throughout their illness and can cause significant psychological distress. This aligns with previous research indicating that fear is a central emotion in cancer diagnosis and adjustment (Pitt et al. 2021).

Our study highlighted that anger, common in cancer diagnosis adjustment (Averill 1982), ranks third in intensity following fear and surprise. Suppressing anger can worsen prognosis, highlighting the importance of addressing and managing this emotion. Strategies to accept and rethink negative emotions are crucial for coping with cancer and other life-threatening illnesses (Chambers et al. 2012).

Attributing significance to cancer experiences, reduces distress (Moye et al. 2020). Promoting spiritual well-being also lessens cancer’s negative impact, while efforts to boost hope continue (Daboui et al. 2018). Our study reveals varying levels of hope among patients, with different levels of hope observed depending on the disease stage. While early-stage patients often hope for healing, those receiving palliative care tend to emphasize spiritual hope (Baczewska et al. 2019). However, we noted limited spiritual hope, among our participants, indicating a potential area for further investigation.

Views have evolved from slow, agonizing deaths linked to sin in medieval times to painless, dignified deaths today (Ariès 1967). Decision-making is tough due to poor communication, conflicting family hopes, and others’ involvement.

In terms of end-of-life care, our study highlighted the importance of factors such as information provision, caregiver trust, and symptom management in achieving a good death for cancer patients (Ferlay et al. 2021). However, we also found that discussions about death are often avoided, indicating a need for improvement in communication and end-of-life planning (Moye et al. 2020).

Health-care providers should focus on fostering open discussions about emotions like fear and anger, as these profoundly influence patients’ decision-making and relationships with caregivers. Training in emotional support and communication is necessary to address these psychological challenges effectively.

### Cultural attitudes and palliative care expectations

Ancient Greeks viewed “Need” as divine, filling gaps (Leeming 2006). Disease, a variant, prompts the addressing of deficiencies due to perceived threats, forming expectations (Ghandourh 2016). Our study revealed misconceptions and challenges surrounding palliative care expectations among cancer patients. Misconceptions hindered decision-making and lowered the quality of life for patients, emphasizing the importance of access to honest information and improved communication in palliative care settings (Weeks et al. 2012).

This study highlights the primary need for information among palliative care patients. The lack of health-related information is explicitly reiterated in the narratives of all participants. Greek cultural attitudes toward death, rooted in Orthodox Christianity and family-centric traditions, shape expectations in palliative care by emphasizing spiritual hope and protective caregiving. Families often shield patients from distressing information, believing it preserves emotional well-being, which can hinder open discussions and care planning. Societal stigma surrounding death further limits honest communication between patients, families, and health-care providers, leading to unmet informational and emotional needs. Many Greeks perceive palliative care as a last resort, focusing narrowly on pain relief rather than holistic support. This finding is consistent with previous studies on unmet needs of cancer patients (Sanson-Fisher et al. 2000). It reveals self-management challenges due to insufficient information, leading to unrealistic expectations. However, more recent research has shown that the greatest needs in this population are related to psychological symptoms. This suggests that there have been improvements in providing information to patients internationally (Li and Girgis 2006; Smith et al. 2007; White et al. 2012). This finding requires further exploration to understand the way and extent to which health-care professionals discuss the disease and its progression in Greece.

Oncologists’ reluctance to share complete information, especially with poor prognoses, exacerbates the issue (Beernaert et al. 2015).

Health-care professionals may avoid open discussions in patient education due to concerns about distress, hope, family wishes, and doctor–patient relationships (Gordon and Daugherty 2003). However, this study underscores the role of information in building trust, addressing anxiety, depression, and hope loss. Patient preferences vary by country, contradicting prior Greek findings and reflecting evolving attitudes. Honest discussions can manage anxiety and depression while maintaining hope (Smith 2015).

Health-care providers should address these cultural challenges by fostering trust and ensuring honest, culturally sensitive communication. Incorporating family dynamics into discussions while respecting patients’ preferences can help bridge informational gaps. Developing culturally tailored guidelines for discussing prognosis and end-of-life care is crucial to improving palliative care.

### Unmet needs in palliative care

Patients have diverse health-care expectations. This study identifies factors fostering hope and improving relationships: cancer knowledge, alternative treatments, symptom control, humor, and not abandoning patients (Masel et al. 2016). Behaviors diminishing hope include nervousness, sharing information with family first, and avoiding patient information (Hancock et al. 2007).

In van Klinken’s research, palliative care patients expected discussions, professional competence, and palliative care knowledge, echoing this study’s focus on patient interactions. Participants in van Klinken’s study also wanted their general physician involved, recognizing palliative care physicians’ expertise, akin to this study’s findings (van Klinken et al. 2020).

In this study, 50% of oncologist-referred participants initially distrusted palliative care, while the other 50% seeking it independently did not. Involving oncologists and caregivers is crucial, as negative attitudes can impact care quality and lead to burnout (Asai et al. 2007).

Male medical students may find palliative care less satisfying than other services, with positive attitudes developing with age (Diver et al. 2018). Limited knowledge about palliative care complicates exploring expectations, highlighting medical personnel’s uncertainty about its functioning (Barclay et al. 2015).

Patients sought palliative care mainly for pain relief, prioritizing psychological support second, warranting further study. Besides pain relief, the need for psychological support is the foremost requirement of these patients, surpassing the need for information in the international literature (Li and Girgis 2006; White et al. 2012). Cancer recurrence is a significant psychological concern. Effective patient discussion relies on health-care professionals’ active listening.

Additionally, 33% expected practical help, such as financial support and future planning, aligning with Masel’s findings regarding palliative care referrals addressing practical needs (Masel et al. 2016).

Health-care systems must enhance interdisciplinary collaboration to provide integrated support, including psychological care and practical assistance. Addressing these unmet needs through tailored interventions and proactive palliative care referrals can improve patients’ quality of life.

## Conclusion

This study highlights the multifaceted nature of hope, emotional responses, and unmet needs in palliative care, particularly within the Greek context. Key findings emphasize the need for improved communication, psychological support, and practical assistance to address these challenges effectively. Recommendations include training health-care providers in patient-centered communication and emotional support, developing guidelines for discussing prognosis and end-of-life care, and enhancing interdisciplinary collaboration to address practical and psychological needs.

Understanding cancer patients’ unmet needs and hopes, and recognizing Palliative Care’s benefits, is crucial for better care. Proactively involving health-care professionals in referring patients to PCUs fosters a holistic, patient-centered approach. This ensures comprehensive assessment and personalized, high-level care beyond medical treatment. Collaboration between patients and providers creates a supportive health-care ecosystem. A proactive, multidimensional approach, blending empathy with medical expertise, enhances cancer patients’ well-being in similar contexts.
